# Fish models for ecotoxicology

**DOI:** 10.1186/1751-0147-54-S1-S14

**Published:** 2012-02-24

**Authors:** Leif Norrgren

**Affiliations:** 1 Department of Biomedicine and Veterinary Public Health, Faculty of Veterinary Medicine and Animal Science, Swedish University of Agricultural Sciences, 750 07 Uppsala, Sweden

## Background

Development of fish models for assessment of chemicals, which may interfere with different parts of the life cycle, act over consecutive generations and with a potential to have impact on populations, are essential for risk assessment and environmental protection.

## 1. Laboratory models

Fish laboratory models covering different parts of the life cycle and different routes of exposure are continuously developed and modified in order to meet new challenges. The most important exposure routes are by injection, trough contaminated water or feed [1]. For laboratory studies zebrafish (*Danio rerio*), medaka (*Oryzias latipes*), fathead minnow (*Pimephales promelas*) and three-spined stickleback (*Gasterosteus aculeatus*) are key species proposed by OECD.

Examples of laboratory models are;

***-Fish Embryo Assays (FEAs)*** are used to study early developmental disorders. Exposure is performed from fertilisation to hatching. A number of developmental parameters are included, i.e. stage, somite formation, heart rate, eye development, pigmentation, tail lifting, hatching, behaviour etc. FEAs have been used to evaluate for instance musks [2], effluents waters from the oil production, pharmaceuticals [3,4] and waste products [5].

***-The Fish Sexual Developmental Test (FSDT)*** has been developed in Nordic countries to evaluate the impact on Endocrine Disrupting Chemicals (EDCs) on sex differentiation and sex ratios. The FSDT has recently been accepted as an OECD guideline. The FSDA has been shown that laboratory fish species show changed VTG concentrations and skewed sex ratios after exposure to estrogens or androgens [6-8].

***-The Fish Screening Assay (FSA)*** is similarly to the FSDA primarily developed to evaluate EDCs. This assay is initiated by a preexpsure period to confirm normal reproductive capacity. Exposure is performed trough water or feed during at least 21 days. The exposure period is followed by evaluation for instance of reproductive fitness, fecundity and fate of the offspring [9,10]

***-Fish Full Life Cycle Assays (FFCAs)*** covers exposure during at least two consecutive generations. FFCAs are the ultimate models to predict risks for populations; however, these long-term exposure models are very expensive to perform and further evaluation is necessary in order to confirm robustness and to define suitable endpoints.

## 2. In situ models

Besides laboratory assays more complex tests can be performed by using in situ exposure models by deployed cages. These models are used to determine for instance offshore oil production, impact of remediation activities [11] and sewage/industrial effluents.

## 3. Mesocosm models

Mesocosm models have the advantage compared to laboratory and in situ models that these maintain a natural ecosystem community close to natural conditions and thereby mimicking a real ecosystem. Mesocosm models can be either land-or water based. Interactions between algae, invertebrates and fish can be assessed.

## Conclusions

Fish models to assess the impact of chemicals are continuously developed in order to introduce more sensitive and relevant endpoints. Today, this development includes a panorama of methodologies such as gene expression, proteomics, physiological biomarkers, pathology, reproduction and behaviour.
